# Effectiveness of extracorporeal shock wave monotherapy for avascular necrosis of femoral head: A systematic review protocol of randomized controlled trial

**DOI:** 10.1097/MD.0000000000015119

**Published:** 2019-04-05

**Authors:** Hua-Yu Tang, Yu Zhao, Yu-Zhi Li, Tian-Shu Wang

**Affiliations:** a Second Ward of Orthopedis Department, First Affiliated Hospital of Jiamusi University, Jiamusi; b Department of Orthopedis, Huludao Central Hospital, Huludao; c Department of Urology, First Affiliated Hospital of Jiamusi University, Jiamusi, China.

**Keywords:** avascular necrosis, effectiveness, extracorporeal shock wave, femoral head, safety, systematic review

## Abstract

**Background:**

Previous clinical studies have reported that extracorporeal shock wave (EPSW) monotherapy can effectively treat avascular necrosis of femoral head (ANFH). However, no systematic review has been conducted to assess its effectiveness and safety for patients with ANFH. Therefore, this study will systematically assess the effectiveness and safety of EPSW monotherapy for patients with ANFH.

**Methods:**

In this study, the following electronic databases will be searched from their inceptions to the present: Cochrane Library, EMBASE, PUBMED, Cumulative Index to Nursing and Allied Health Literature, China National Knowledge Infrastructure, and Chinese Biomedical Literature Database. This study will include randomized controlled trials for assessing the effectiveness and safety of EPSW monotherapy for patients with ANFH. Two independent authors will perform study selection, data extraction, and methodology assessment. RevMan 5.3 software will be used for statistical analysis.

**Results:**

This systematic review will provide latest summary evidence of EPSW monotherapy for patients with ANFH through assessing the outcome measurements. The primary outcome is pain intensity, which can be measured by visual analog scale or relevant measurement tools. The secondary outcomes are functional status of attacked femoral head, as assessed by Western Ontario and McMaster Universities Osteoarthritis Index, or other relevant scales; quality of life, as evaluated by The 36-Item Short Form Health Survey, or related instruments; and adverse events.

**Conclusion:**

The results of this study may provide the latest evidence for assessing the effectiveness and safety of EPSW for the treatment of ANFH.

**Dissemination and ethics:**

This study does not require ethical approval, because no individual data will be involved in this systematic review. The findings of this study will be published through a peer-reviewed journal.

**Systematic review registration:**

PROSPERO CRD42019124665.

## 1 Introduction

Avascular necrosis of femoral head (ANFH) is a very common progressive orthopedic disorder.^[1–3]^ This condition often manifests as severe pain, limitation of lower limbs activity.^[4–6]^ It mainly affects patients those aged between 20 to 50 years old.^[7]^ Many factors are responsible for this disorder, such as diabetes, hypertension, vasculitis, pancreatitis, and so on.^[8–13]^ Thus, effective managements for this disorder are very important and necessary. Otherwise, it may lead to poor quality of life in patients who suffer from this disorder.^[14–16]^

Extracorporeal shock wave (EPSW) has been reported to treat a variety of orthopedic disorders effectively, such as chronic rotator cuff tendonitis, rheumatoid arthritis, chronic plantar fasciitis, and ANFH, especially for ANFH.^[17–19]^ Lots of clinical trials have reported that EPSW has been used for ANFH treatment, and has achieved a promising effectiveness.^[20–28]^ However, up to present, no study systematically has investigated the effectiveness and safety of EPSW for ANFH. Therefore, this systematic review will assess the effectiveness and safety of EPSW for patients with ANFH.

## 2 Methods and analysis

### 2.1 Study selection criteria

#### 2.1.1 Types of studies

Only randomized controlled trials (RCTs) evaluating the effectiveness and safety of EPSW for ANFH will be considered for inclusion in this study. The other studies, such as non-RCTs, non-controlled trials, non-clinical studies, and quasi-RCTs will be excluded in this study.

#### 2.1.2 Types of participants

Patients with clinically diagnosed with ANFH will be fully considered for inclusion with any restrictions, such as race, age, and gender.

#### 2.1.3 Types of treatments

In the experimental group, only EPSW monotherapy will be considered for inclusion. In the control group, any kinds of interventions can be used, except any forms of EPSW alone or combination.

#### 2.1.4 Types of outcome measurements

The primary outcome is pain intensity. It can be measured by any pain instruments, such as visual analog scale.

The secondary outcomes are functional status of attacked femoral head, as measured by any relevant scales, such as Western Ontario and McMaster Universities Osteoarthritis Index; quality of life, as evaluated by any associated measurement tools, such as The 36-Item Short Form Health Survey; and any adverse events.

### 2.2 Search strategy

Cochrane Library, EMBASE, PUBMED, Cumulative Index to Nursing and Allied Health Literature, China National Knowledge Infrastructure, and Chinese Biomedical Literature Database will be searched from their inceptions to the present without any language restrictions. Reference lists of relevant studies will also be searched to avoid missing any potential eligible RCTs. The search strategy for Cochrane Library has been developed in consultation with an experienced librarian and is shown in Table 1. This search strategy will also be adapted and applied to other databases.

**Table 1:**
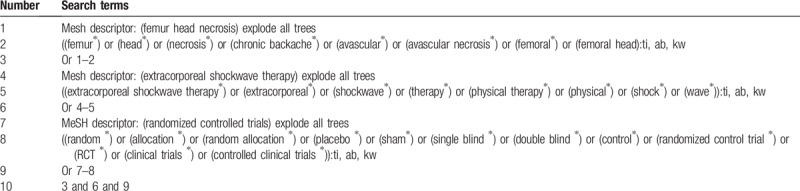
Search strategy applied in Cochrane Library database.

### 2.3 Study selection

Two authors will independently scanning the title, or/and abstract initially. Full texts will be further read if insufficient information can be utilized for judgment by screening title and abstract only. All disagreements regarding study selection have arisen between 2 authors, and a third experienced author will help to solve them by discussion. This study has been reported according to the Preferred Reporting Items for Systematic Reviews and Meta-Analyses (PRISMA)^[29]^ and PRISMA-Protocol guidelines.^[30,31]^ Additionally, a PRISMA flow chart will be utilized to provide transparency of number of studies with specific inclusion and exclusion at each stage.

### 2.4 Data extraction and management

Two authors will independently extract data according to the pre-designed data extraction sheet. The sheet includes the following information: title, first author, year of publication, country, funding information, setting, study design and methods (such as randomization, blinding, and concealment), sample size, dosage, treatment frequency, and duration, and all primary, secondary and other outcome measurements. If any divergences regarding the data extraction between 2 authors exist, a third experienced author will be consulted and settle them down through discussion.

### 2.5 Risk of bias assessment

Risk of bias assessment will be assessed by using Cochrane Handbook of Systematic Review of Interventions. It comprises of 7 fields, and each field is categorized as a high risk of bias, unclear risk of bias, or low risk of bias. Two authors will independently conduct the risk of bias assessment. A third experienced author will act as an arbiter through discussion if any disagreements arise between 2 authors.

### 2.6 Statistical analysis

#### 2.6.1 Treatment effect measurement

The risk ratio and 95% confidence intervals (CIs) will be presented for dichotomous data, while the mean difference, or standardized mean difference and 95% CIs will be expressed for continuous data.

#### 2.6.2 Heterogeneity evaluation

The test of *I*^2^ will be utilized to check the heterogeneity among included studies. There is reasonable heterogeneity among those included studies if *I*^2^ ≤50%. On the other hand, there is substantial heterogeneity among those include studies if *I*^2^ >50%.

#### 2.6.3 Data synthesis

If acceptable heterogeneity will be detected, a fixed-effect model will be used to pool the data, and meta-analysis will be conducted. On the other hand, if substantial heterogeneity will be identified, a random-effect model will be used to pool the data. Meanwhile, subgroup analysis will be performed to detect any reasons that may cause significant heterogeneity. If there is acceptable heterogeneity after the subgroup analysis, meta-analysis will be conducted. Otherwise, there is still substantial heterogeneity after the subgroup analysis, meta-analysis will not be conducted, and a narrative description will be elaborated instead.

#### 2.6.4 Subgroup analysis

If heterogeneity is substantial among those included studies, subgroup analysis will be performed in accordance with different characteristics, treatments, controls, and outcome instruments.

#### 2.6.5 Sensitivity analysis

Sensitivity analysis will be carried out to check the robustness of pooled results by removing low quality of studies.

#### 2.6.6 Reporting bias

Funnel plot and Egger test will be conducted to check any possible of the reporting bias if more than 10 eligible RCTs are included in this study.

## 3 Discussion

This systematic review is the first study to investigate the effectiveness and safety of EPSW monotherapy for patients with ANFH. Its findings will supply a detailed summary of the up-to-date evidence relevant of EPSW in pain relief, improvement of functional status of attacked femoral head, and enhancement of quality of life in patients with ANFH. This evidence may be helpful to clinician, patients, as well as the health policy makers regarding the use of EPSW for the treatment of ANFH.

## Author contributions

**Conceptualization:** Hua-Yu Tang, Tian-Shu Wang.

**Data curation:** Hua-Yu Tang, Yu Zhao, Yu-Zhi Li.

**Formal analysis:** Yu Zhao, Tian-Shu Wang.

**Funding acquisition:** Hua-Yu Tang.

**Investigation:** Hua-Yu Tang, Tian-Shu Wang.

**Methodology:** Yu Zhao, Yu-Zhi Li.

**Project administration:** Hua-Yu Tang, Tian-Shu Wang.

**Resources:** Yu Zhao, Yu-Zhi Li, Tian-Shu Wang.

**Software:** Yu Zhao, Yu-Zhi Li.

**Supervision:** Tian-Shu Wang.

**Visualization:** Hua-Yu Tang, Yu-Zhi Li, Tian-Shu Wang.

**Writing - Original Draft:** Hua-Yu Tang, Yu Zhao, Yu-Zhi Li, Tian-Shu Wang.

**Writing - Review & Editing:** Hua-Yu Tang, Yu Zhao, Yu-Zhi Li, Tian-Shu Wang.
